# Shared and Species-Specific Patterns of Nascent Y Chromosome Evolution in Two Guppy Species

**DOI:** 10.3390/genes9050238

**Published:** 2018-05-03

**Authors:** Jake Morris, Iulia Darolti, Natasha I. Bloch, Alison E. Wright, Judith E. Mank

**Affiliations:** 1 Department of Genetics, Evolution and Environment, University College London, London WC1E 6BT, UK; iulia.darolti.15@ucl.ac.uk (I.D.); n.bloch@ucl.ac.uk (N.I.B.); judith.mank@ucl.ac.uk (J.E.M.); 2 Department of Animal and Plant Sciences, University of Sheffield, Sheffield S10 2TN, UK; a.e.wright@sheffield.ac.uk; 3 Department of Organismal Biology, Uppsala University, 752 36 Uppsala, Sweden

**Keywords:** sex chromosomes, Y-chromosome, heterochromatin, *Poecilia*, guppies, sex determination, pigmentation

## Abstract

Sex chromosomes form once recombination is halted around the sex-determining locus between a homologous pair of chromosomes, resulting in a male-limited Y chromosome. We recently characterized the nascent sex chromosome system in the Trinidadian guppy (*Poecilia* *reticulata*). The guppy Y is one of the youngest animal sex chromosomes yet identified, and therefore offers a unique window into the early evolutionary forces shaping sex chromosome formation, particularly the rate of accumulation of repetitive elements and Y-specific sequence. We used comparisons between male and female genomes in *P. reticulata* and its sister species, Endler’s guppy (*P. wingei*), which share an ancestral sex chromosome, to identify male-specific sequences and to characterize the degree of differentiation between the X and Y chromosomes. We identified male-specific sequence shared between *P. reticulata* and *P. wingei* consistent with a small ancestral non-recombining region. Our assembly of this Y-specific sequence shows substantial homology to the X chromosome, and appears to be significantly enriched for genes implicated in pigmentation. We also found two plausible candidates that may be involved in sex determination. Furthermore, we found that the *P. wingei* Y chromosome exhibits a greater signature of repetitive element accumulation than the *P. reticulata* Y chromosome. This suggests that Y chromosome divergence does not necessarily correlate with the time since recombination suppression. Overall, our results reveal the early stages of Y chromosome divergence in the guppy.

## 1. Introduction

Sex chromosomes, where chromosome complement determines whether an individual develops as a male or female, have evolved independently in many diverse lineages [1,2]. Sex chromosomes form when recombination is halted between homologous chromosomes around the sex determining-locus [3,4,5,6,7]. Although there are notable exceptions to the general pattern of sex chromosome function and evolution [8,9,10], many independently evolved sex chromosomes exhibit similar evolutionary signatures [1], making them one of the most common cases of genomic convergence. In addition to permitting X and Y chromosome divergence, the loss of recombination for the male-specific Y chromosome leads to a range of evolutionary processes, including rapid loss of gene activity, high rates of pseudogenization, and accumulation of repetitive elements [7,11,12,13,14]. These processes can lead to major differences in size and gene content between the X and Y, and in old, highly heteromorphic sex chromosome systems such as those in *Drosophila* and therian mammals, the Y chromosome is largely composed of repetitive elements with only a handful of genes [7,12,13].

Y chromosomes have been notoriously difficult to sequence for two key reasons. First, Y chromosomes are present in only one copy within the genome, requiring double the read depth of diploid regions of the genome for the same coverage without some mechanism of enrichment [15]. Additionally, the accumulation of repetitive DNA complicates complete assemblies, particularly from short-read sequencing data. These difficulties have historically limited studies of Y chromosomes to model systems, such as mammals [13,16], *Drosophila* [7], and a few other groups [17,18,19]. Importantly, although these models reveal a great deal about highly degenerate Y chromosomes, they tell us very little about the earliest stages of Y divergence. Only recently have the initial stages of sex chromosomes formation been studied using genomic approaches [20,21,22,23,24,25,26,27,28,29]. These nascent sex chromosome systems offer the opportunity to elucidate the first stages of sex chromosome differentiation, and study the rate and process of Y divergence.

The species within the family Poeciliidae are live bearing fish, many with pronounced sexual dimorphism. Within *Poecilia*, the Trinidadian guppy (*Poecilia reticulata*) and its sister species Endler’s guppy (*Poecilia wingei*) diverged approximately 3–4 Mya [30]. Previous cytogenetic work has shown that the Y chromosome has a large pseudoautosomal region, along with a smaller heterochromatin block, which is variable in size between populations [31]. Interestingly, a particularly large heterochromatic block has been found in some populations of *P. wingei* [31], making the Y chromosome the largest chromosome in the genome in this species.

We recently characterized intra-specific variation in X–Y divergence in *P. reticulata*. Overall, our results indicate a recent origin of the Y chromosome in this system, with an expansion of the non-recombining region in some Trinidad populations in response to elevated levels of sexual selection [32]. The recent origin and intra-specific diversity of the guppy Y chromosome offers an important opportunity for studying the initial stages of Y chromosome differentiation. Additionally, many sexually antagonistic male coloration loci have been inferred to be Y linked in the guppy [33,34], offering great potential to elucidate the role of sexual conflict, where an allele benefits one sex at the expense of the other, in sex chromosome formation [3]. Because of this, the gene content of the guppy Y chromosome is of particular interest.

*k*-mer analysis has been particularly useful in recent studies of the Y chromosome. *k*-mers are all possible sub-sequences of a given length, in this case within a genome. New in silico methods for identifying Y-specific sequences in both model and non-model systems [35,36] have now begun to exploit the fact that male-specific *k*-mers are likely to be Y-linked, based on the fact that the Y chromosome is only present in males. Through the identification of these *k*-mers, we can now find and assemble Y-reads into contigs and use these to identify sex-linked sequences and gene content. This approach has been used to characterize young sex-specific regions in persimmon [37], date palm [38], and basket willow [22].

Comparisons of male and female genomes not only make it increasingly possible to characterize the male-specific non-recombining region of the Y chromosome in both nascent [37,38] and highly heteromorphic [15,39] sex chromosome systems, but also offer key insights into the processes that shape Y chromosome genomes and gene content. In theory, the insertion of repetitive sequences on the Y chromosome or major differences in size of the sex chromosomes would be predicted to lead to differences in the *k*-mer profile between males and females, and this approach can reveal the amount of repetitive element accumulation on a newly emerged Y chromosome [22].

Here, we used *k*-mer analysis to compare male and female genomes in both *P. reticulata* and its sister species *P. wingei* (Endler’s guppy), which share an ancestral sex chromosome [31] located on linkage group 12 of the genome [40,41]. We first assembled male-specific *k*-mers shared in both species (*Y*-mers) into contigs, uncovering substantial homology to the X chromosome, consistent with only recent recombination suppression between the two chromosomes. From this *Y*-mer assembly, we then identified a significant enrichment of genes that have been implicated in pigmentation, as well as two plausible candidate genes that may be involved in sex determination. Our *k*-mer analysis also reveals very low levels of repetitive element accumulation on the *P. reticulata* Y chromosome overall, indicating only a small region with significant divergence from the X chromosome. In contrast, the Y chromosome of *P. wingei* exhibits a greater aggregation of repetitive elements, consistent with the enlarged heterochromatic block observed in the non-recombining region in this taxon [31].

## 2. Materials and Methods

### 2.1. Sample Collection and Sequencing

We resequenced the DNA from tail tissue of two male and two female *P. reticulata* samples from a large outbred population established in 1998. We also resequenced tail tissue from three male and three female *P. wingei* derived from wild stock and maintained for >20 years in captive populations by a fish fancier (St Albans, UK). All fish were euthanized and sampled according to national and institutional ethical guidelines. DNA was extracted with a DNeasy Blood and Tissue Kit (Qiagen, Hilden, Germany) using the manufacturer protocols. We sequenced DNA using an Illumina HighSeq 4000 (Illumina, San Diego, CL, USA) at The Wellcome Trust Centre for Human Genetics, University of Oxford, UK using standard protocols, resulting in an average of 269 million 100 bp paired-end reads per *P. reticulata* sample (data available from NCBI Sequencing read Archive; Bioproject ID PRJNA353986) and an average 244 million 150 bp paired-end reads per *P. wingei* sample (European Nucelotide Archive; ID PRJEB26489).

### 2.2. Quality Trimming and Filtering

We quality and adaptor trimmed reads using Trimmomatic v0.36 [42], with bases trimmed if the sliding window average Phred score over four bases was <15 or if the leading/trailing bases had a Phred score <3. The minimum length for reads was set to 36 bases *P. reticulata* and to 50 for the longer *P. wingei* reads. We then corrected sequencing errors with Lighter v1.1.1 [43] using a *k*-mer size of 31.

### 2.3. k-mer Counting

One advantage of this *k*-mer counting approach is the method’s robustness to differences in read length and filtering between our two guppy species. We first pooled processed reads by sex without normalization. We then used Jellyfish v2.2.6 [44] to count 31 bp *k*-mers canonically (*k*-mer and reverse compliment treated as the same) with no bloom filter or high or low cut-off, so that we counted all *k*-mers, including those with low coverage. Choosing *k*-mer size is a balance between ensuring a high proportion of unique *k*-mers between males and females, at the cost of increased error and the computational requirements needed to have lower repetition within the dataset. [35]. Previous studies have shown that *k*-mer sizes below 18 are problematic [36] and that the optimal *k*-mer size ranges from 21 bp to 31 bp. To output *k*-mer and their respective counts we used the Dump command, while we used the Histo command to count the number of *k*-mers at each coverage.

### 2.4. Identifying Y-Sequence

We based Y-sequence identification on the Dump outputs (Jellyfish v2.2.6) for both the *P. reticulata* and *P. wingei* analyses of each sex. From these we used custom scripts (Supplementary File S2) to assemble a table for each species composed of each *k*-mer sequence and its corresponding male and female read count. We then identified unique male and female *k*-mers in each species. In order to identify the ancestral Y chromosome regions, we identified male unique *k*-mers shared in both species and with normalized coverage >30× (the threshold at which we could detect an excess of male unique *k*-mers relative to female unique *k*-mers) and designated them as *Y*-mers. We then used these *Y*-mers to extract reads and their paired sequence from the male *P. reticulata* samples, which we then used to build an assembly using ABySS v1.9.0 [45] with default settings and K = 15.

### 2.5. Y-Sequence Characterization

We first BLASTed all contigs >100 bp with BLASTn [46] to the female *P. reticulata* genome [47] to identify homology using the BLAST hit with the highest *e*-value (provided it was above an *e*-value threshold of 0.00001). We also BLASTed each contig to the NCBI nucleotide collection with MegaBLAST to identify predicted genes and used this to compile a list of putative Y genes that matched at least one of our contigs (again using an *e*-value threshold of 0.00001). This list only contained genes where the BLAST hit was unambiguously to that gene. Therefore, in cases where a contig hit multiple different predicted genes in the database (not counting orthologs), the gene was only included if the difference in the bit-score for the top two hits was >30%. This conservative filtering step removes instances where a single contig had multiple hits to different, seemingly unrelated genes, which complicated inference of function. We also checked for genes in our list annotated to pigmentation (GO:0043473) in *Danio rerio* and then tested for significant enrichment of these pigmentation genes using a one-tailed Χ^2^ test with Yate’s correction.

### 2.6. Origins of Y Genes

Finally, we wanted to investigate whether our putative Y genes were ancestrally shared between the X and Y, or whether many may have been moved to the Y via translocation. To do this, we took the DNA sequence of our putative Y genes and BLASTed these (with MegaBLAST) to the female *P. reticulata* reference genome in order to identify their genomic position. Where a gene was present in our list from two species (multiple orthologs), we used the ortholog sequence from the species most closely related to *P. reticulata*. Again we used the >30% bit-score threshold to decide whether a hit was unambiguously hit to a given scaffold.

### 2.7. K-mer Composition Comparisons

We used Histo analyses from Jellyfish v2.2.6 to make *k*-mer composition comparisons. We normalized coverage for each set of pooled individuals by the total basepairs remaining after trimming. Furthermore, the Y chromosome in *P. wingei* has been shown cytologically to contain a large heterochromatic block, consistent with the accumulation of large amounts of repetitive elements [31], and the Y chromosome in this species is demonstrably larger than the X, and is in fact the largest chromosome in the genome. The large size of the Y chromosome could affect our comparisons, as sequencing depth for the remainder of the genome would be reduced in males. In order to normalize male and female genomes for comparison, we calculated the total basepairs of sequence composed of male and female unique *k*-mers. We then calculated the total excess base pairs composed of male unique *k*-mers, and normalized female counts by this proportion.

## 3. Results

### 3.1. Y-mer Identification

**Figure 1:**
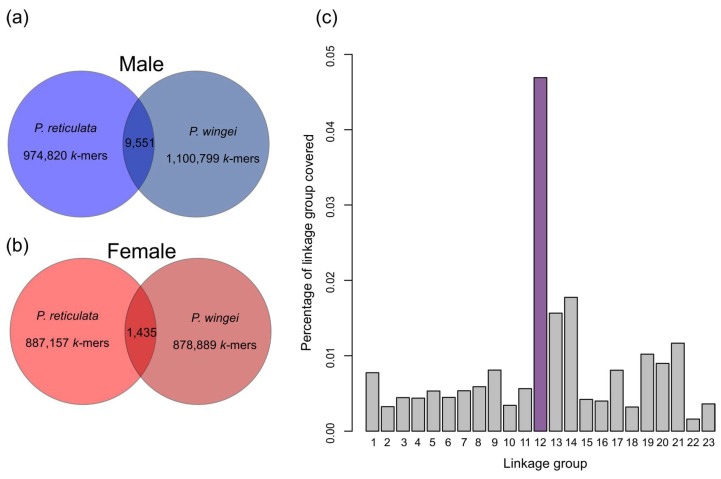
(**a**) Male-specific *k*-mer counts above 30× coverage in *Poecilia reticulata* and *P. wingei* and shared across both; (**b**) female-specific *k*-mer counts above 30× coverage in *P. reticulata* and *P. wingei* and shared across both; (**c**) homology of assembled Y-linked contigs to each linkage group in the *P. reticulata* genome, showing the percentage of each linkage group covered by contigs ((bp length of contigs/bp length of linkage group) × 100).

It is possible to identity Y-specific sequence by identifying *k*-mers that are specific to males (*Y*-mers). Using this approach, we observed an excess of male-specific *k*-mers above ~30× coverage in both *P. reticulata* and *P. wingei* (Supplementary Figure S1), consistent with small but detectable amounts of male-specific sequence on the Y in both species. Because the sex chromosomes in *P. wingei* and *P. reticulata* are orthologous [31], we identified *Y*-mers shared by both species [38] in order to assemble the ancestral Y chromosome. We identified 9551 *Y*-mers with >30× coverage that were shared by both species (Figure 1). These were then assembled into 550 contigs, with a mean length of 167 bp (range 100–1051 bp), totaling 91.908 Kb of putative male-specific sequence.

In theory, divergent regions of putative Y sequence should show little homology to the *P. reticulata* female reference genome [47]. Indeed, 162 of these contigs, totaling 25.775 Kb of sequence showed the greatest homology to 134 unplaced genomic scaffolds, while 38 contigs, totaling 5.671 Kb, did not show significant homology (*e*-value < 0.00001) to any scaffold in the *P. reticulata* genome. Less divergent regions of putative Y sequences should bear some homology to the X chromosome. Importantly, 12.821 Kb, or 21.20% of the remaining putative Y scaffolds with some homology to mapped scaffolds in the female genome assembly showed the greatest sequence similarity to linkage group 12 (Figure 1), which has previously been identified as the sex chromosome [32].

### 3.2. Characterization of the Y Sequence

Of the 550 Y-linked contigs that we assembled, 241 showed homology to the NCBI nucleotide database. The majority of these 241 hits were to uncharacterized loci, but we also identified 40 characterized genes (Table 1). Our putative list of Y genes included three implicated in pigmentation (*MLPH*, *RAB27B*, and *Kita*) in *D. rerio*, a significant enrichment in our list (*p* = 0.001). Furthermore, we identified two genes in our list that are plausible candidate sex determining genes (*MED13L* and *CYP27A*).

### 3.3. Origins of Y Genes

By looking at the genomic position of our putative Y genes in the female *P. reticulata* genome, we were able to identify eleven genes that are ancestrally shared between the X (linkage group 12) and the putative Y (Table 1). A further seven genes were found to have multiple hits across the genome, making it impossible for us infer their ancestry, while eight were found on unplaced scaffolds, meaning that their ancestral location in the genome was unknown. This left fourteen genes that appear to be ancestrally autosomal.

### 3.4. Poecilia reticulata k-mer Composition

**Figure 2:**
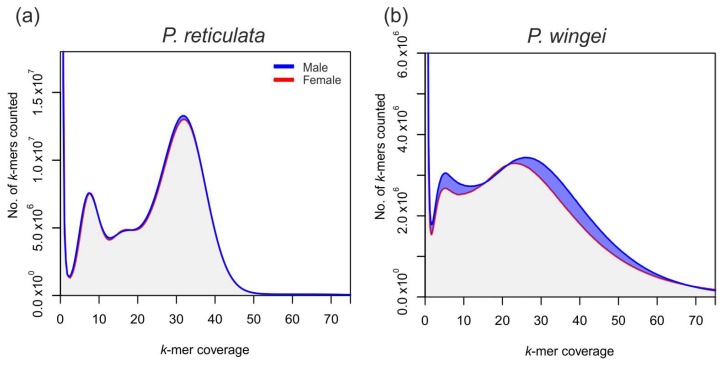
Male (blue) and female (red) *k*-mer coverage profiles in (**a**) *P. reticulata* and (**b**) *P. wingei*.

The accumulation of repetitive sequences on the Y chromosome, or major differences in size of the sex chromosomes, will create differences in the *k*-mer profile between males and females. We found that in *P. reticulata*, males and females showed only a small difference in *k*-mer composition (Figure 2a). As expected, we found the highest peak in both sexes to be in the number of single copy *k*-mers, which likely represent sequencing errors. There were three further peaks in *k*-mer coverage, at ~8×, ~16× and ~32× coverage. The first of these likely represents alleles found at a frequency of 25%, the second peak represents alleles found at a frequency of 50%, and the last represents alleles found at a frequency of 100%. There was a slight increase in male *k*-mer coverage at this third peak, suggesting only a few distinct differences between the X and Y chromosome in this species, and overall low levels of accumulation of repetitive elements on the Y chromosome.

### 3.5. Poecilia wingei k-mer Composition

The number of *k*-mers was far lower in *P. wingei* than in *P. reticulata*, despite analyzing an additional sample of each sex. This suggests much lower levels of genetic diversity in our *P. wingei* population, possibly due to inbreeding. Similar to *P. reticulata*, the highest peak in both sexes was in the number of single-copy *k*-mers. There were two further peaks in *k*-mer coverage at ~5–6× and ~20–30× coverage. The first of these likely represents alleles found at a frequency of 17%, while the second represents alleles found at a frequency of 100%. In sharp contrast to *P. reticulata*, the *k*-mer profiles in *P. wingei* show greatly increased *k*-mer coverage in males at these two peaks. The lack of the middle peak in *P. wingei*, that in *P. reticulata* represented alleles at a frequency of 50%, is likely due the use of three *P. wingei* individuals, and to the overall lower diversity found in *P. wingei*. However, the region of ~10–12× coverage did also show increased *k*-mer coverage in males. The mean coverage at the homozygous peak was also highest in males. Altogether, this data supports the hypothesis that there is increased accumulation of repetitive elements on the young Y chromosome of *P. wingei*.

## 4. Discussion

### 4.1. Young Sex Chromosomes and Mixed Sources of Genic Content

Our analysis of *Y*-mers in *P. reticulata* and *P. wingei* [31] revealed assembled contigs with substantial homology to linkage group 12, which has previously been shown to be the sex chromosome [32,41]. This is consistent with recent recombination suppression and divergence of the Y from the X chromosome. Our data indicate a small ancestral non-recombining region of the Y chromosome conserved across both species. We also investigated the ancestral origins of the genes that we identified from our *Y*-mers and that we hypothesized to be from this region. Empirical evidence regarding the origins and evolution of Y-linked genic content currently shows conflicting evidence for their ancestral sources in different taxa [48]. In mammals, genes on the Y chromosome have been found to be homologous to the X chromosome, and are found at very low density [6,12,49]. This is consistent with the idea that the heterozygous sex chromosome genic content mostly evolves through gene loss, leading to a depletion of genes that were originally ancestral to that chromosome. In contrast, the genic content of the Y chromosomes of *Drosophila* species show low conservation of genic content compared to the autosomes, with a clear tendency for gene gain on the Y [7,48]. However, a suite of Y-linked genes been extensively characterized in a small number of non-model species [26,50,51]. Of the 26 putative Y genes that we were able to identify a clear homolog for in guppies, we found just under half to be ancestral to the X, while a further 14 appeared to have autosomal homologs. This is consistent with the idea that during the early stages of Y chromosome differentiation, the majority of genes are ancestral to the sex chromosome linkage group, but that genes can also be gained by the Y via tandem or ectopic duplication events from the autosomes [7,50,51,52,53,54], a process that has the potential to rapidly resolve sexual conflict [52,55].

Next generation sequencing coverage can vary substantially within genomes. While our approach will enrich for male-specific *k*-mers, not all the *k*-mers in our *Y*-mer pool are indeed Y-linked, as evidenced by the smaller number of female-specific *k*-mers identified (Figure 1b). However, our analysis does suggest that the vast majority of our inferred *Y*-mers are indeed Y-linked for two key reasons. First, our assembled *Y*-mer contigs preferentially map to linkage group 12 (Figure 1c), which has been previously shown to be the sex chromosome in this system [29,39]. Additionally, we observe a much larger pool of inferred *Y*-mers than inferred female-specific *k*-mers (Figure 1a,b).

### 4.2. Non-Linear Degeneration of the Guppy Y Chromosome

The major differences in *k*-mer profiles (Figure 2) between *P. reticulata* and *P. wingei* reveal that Y chromosome degeneration may not be a linear process that scales with time since recombination suppression. This is consistent with previous work on a range of species [9,10,56].

Large-scale expansions of repetitive elements may be important in heterochromatin formation, and the greater *k*-mer coverage in *P. wingei* males (Figure 1b) is consistent with the heterochromatin block observed on the *P. wingei* Y chromosome from cytogenetic work [31]. The expansion of repetitive sequence in *P. wingei* is far more pronounced than in *P. reticulata* where there is only a small difference in *k*-mer profile between the male and female genomes (Figure 1a). This contrast between our two guppy species has interesting implications for early Y chromosome evolution, suggesting that the accumulation of repetitive elements during sex chromosome evolution is not linear, and therefore not directly correlated with time since recombination suppression. The expansion on the *P. wingei* Y chromosome may have occurred either through enlargement of the non-recombining region or the accumulation of repetitive sequence within the ancestral shared region [31]. It is not possible with these data to differentiate these scenarios, but this provides an interesting area for future work.

### 4.3. Possible Pigmentation Loci Candidates

Out of the 40 putative Y genes, we found three linked to pigmentation in the zebrafish, *D. rerio*, representing a significant-enrichment in our Y gene assembly. One of the putative Y genes we identified was *Kita*, a Tyrosine-protein kinase involved in the kit signalling pathway in our list of Y genes. *Kita* is involved in melanocyte morphogenesis [57], and a non-functional *Kita* gene is the ultimate cause of the guppy *Golden* morph [58]. While we could not identify the ancestral source of *Kita*, we found that the melanophilin-like gene was most likely ancestrally autosomal, while the *RAB27B*-like gene appears to be ancestral to the X chromosome. Both of these genes are linked to melanosome transport. Previous research has shown a number of male color loci linked to the Y chromosome [33,34]. If we are correct that the ancestral source of melanophilin-like gene is autosomal, then this raises the prospect that sexual conflict in guppies over color may be being resolved through duplications of pigmentation genes to the non-recombining portion of the Y chromosome. This would allow selection to independently optimize male coloration functions without adversely affecting other functions necessary in both males and females [55], and would also explain the surprisingly high incidence of Y-linkage of male color traits in guppies [33]. Furthermore, this accumulation of color genes would drive the expansion of the non-recombining region [3,4,32,49,59] between the guppy sex chromosomes.

### 4.4. Possible Sex Determination Candidates

Two genes that are possible candidates for a role in sex determination were also found among our 40 putative Y genes. A *MED13L*-like contig maps to linkage group 12 in *P. reticulata* and belongs to the Mediator complex subunit 13 family of genes. *MED13* plays a role in the androgen receptor signaling pathway and therefore the growth and development of male reproductive organs [60,61].

*CYP27A* does not map to a defined linkage group, and is a cytochrome P450 enzyme. *CYP27A* is known to play a role in both the bile acid biosynthetic process and the sterol metabolic process [62]. In humans, *CYP27A* is found in the human mitochondrial genome, and is known to produce all 27-hydroxycholesterol, which plays a role in the tissue-specific modulation of estrogen receptors [63]. Estrogen receptors are known to play an important role in sexual maturation and have been shown to be involved in sex reversal in alligators [64]. Two members of the cytochrome P450 family, *CYP17* and *CYP19*, have been found to be involved in gonad development in frogs and have been hypothesized to be important for sex determination [65,66].

For *CYP27A* to be a plausible sex-determining gene, it must be located on the Y, and not the mitochondrial genome. We confirmed that this gene is nuclear rather than mitochondrial, BLASTing the *Austrofundulus limnaeus* contig, which contains the ortholog of *CYP27A* to the *P. reticulata* mitochondrial genome [67]. We recovered no evidence of homology, suggesting that the unmapped scaffold in the *P. reticulata* genome containing *CYP27A* is indeed located within the nucleus.

## 5. Conclusions

Our results are consistent with the hypothesis that both *P. reticulata* and its sister species *P. wingei* share an ancestral sex chromosome located on linkage group 12 of the genome. Our *k*-mer analysis reveals very low levels of repetitive element accumulation on the *P. reticulata* Y chromosome overall, while the Y chromosome of *P. wingei* exhibits a greater aggregation of repetitive elements. We describe a number of genes that may play a role in pigmentation, as well as two loci, assembled from reads containing *Y*-mers conserved across both guppy species, that show intriguing similarity to genes that are in families with members that play roles in reproductive development and sex determination. These two genes are thus plausible candidates for a role in guppy sex determination, and provide a useful starting point for further work to identify the locus of sex determination in guppies.

## Figures and Tables

**Table 1:** Putative Y-linked genes and their positions in the *Poecilia reticulata* genome.

Gene	Genome Position
*DNAJC1* ^2^	Unpl. Scaff.
*CAMTA2* ^4^	Unpl. Scaff.
*CDK5R1-like* ^1^	LG8
*CECR5-like* ^1^	Multi. hits
*CNRIP1-like* ^3^	LG1
*CYP27A* ^6^	LG12
*jockey/pol-like* ^2^	Multi. hits
*ELF2-like* ^5^	Multi. hits
*EPHB4-like* ^2^	Unpl. Scaff.
*gastrula zinc finger protein XlCGF17.1-like* ^3^	Multi. hits
*GSN* ^1,5^	Unpl. Scaff.
*KCNV2* ^2^	LG12
*LRRC39* ^1^	LG4
*MDH2* ^1^	LG16
*MED13LL* ^1^	LG12
*MLPH-like* ^1^	LG2
*NFIL3* ^1^	LG12
*NLRC3-like* ^5^	LG12
*nonfunctional kita gene* ^1^	Multi. hits
*OR6N2-like* ^1^	Multi. hits
*PARP4-like* ^1^	LG2
*PDE8B* ^3^	LG12
*RAB27B-like* ^1^	LG12
*RASGRF2-like* ^1,2^	LG12
*RSU1-like* ^4^	LG20
*S1PR3-like* ^4^	LG12
*SAPCD2* ^1^	LG12
*SNAPC4* ^1^	Unpl. Scaff.
*stonustoxin subunit alpha-like* ^4^	LG14
*transposon Helitron gene-like* ^5^	Unpl. Scaff.
*TRIM16-like* ^1,5^	LG17
*TRIM39-like* ^6^	Multi. hits
*TTF1* ^1^	Unpl. Scaff.
*UFM1* ^1^	LG21
*UGT2B31-like* ^1^	LG18
*uncharacterized protein K02A2.6-like* ^2,5^	LG7
*XYLB* ^4^	Unpl. Scaff.
*ZBED1-like*^4^	LG12
*ZCCHC18-like*^2^	LG9
*ZNF146* ^4^	LG2

^1^ *P. reticulata*; ^2^ *P. formosa*; ^3^ *P. latipinna*; ^4^ *P. mexicana*; ^5^ *Xiphophorus maculatus*; ^6^ *Austrofundulus limnaeus*.

## Supplementary Materials

The following are available online at http://www.mdpi.com/2073-4425/9/5/238/s1, Figure S1: Histograms showing counts (log-scaled axis) of male (blue) and female (red) specific *k*-mers at different (normalised) coverages. Bin sizes are of 100× (main) and 30× (inset). Note the excess of male-specific *k*-mers above ~30× coverage in both *P. reticulata* (panel A) and *P. wingei* (panel B). File S1: Excel workbook—Table S1 shows metrics for best BLAST hit for all 241 putative Y contigs with homology to the NCBI nucleotide database; Table S2 shows the 58 contigs (and lengths) with hits to our list of 40 putative Y genes. File S2: Zip drive with shell and python scripts needed to make a male and female *k*-mer coverage table from Jellyfish Dump files.

## References

[1] Bachtrog D., Mank J.E., Peichel C.L., Kirkpatrick M., Otto S.P., Ashman T.-L., Hahn M.W., Kitano J., Mayrose I., Ming R. (2014). Sex determination: Why so many ways of doing it?. PLoS Biol..

[2] Beukeboom L.W., Perrin N. (2014). The Evolution of Sex Determination.

[3] Rice W.R. (1987). The accumulation of sexually antagonistic genes as a selective agent promoting the evolution of reduced recombination between primitive sex chromosomes. Evolution.

[4] Wright A.E., Dean R., Zimmer F., Mank J.E. (2016). How to make a sex chromosome. Nat. Commun..

[5] Charlesworth B. (1991). Evolution of sex chromosomes. Science.

[6] Rice W.R. (1996). Evolution of the Y sex in animals: Y chromosomes evolve through the degeneration of autosomes. Bioscience.

[7] Koerich L.B., Wang X., Clark A.G., Carvalho A.B. (2008). Low conservation of gene content in the *Drosophila* Y. chromosome. Nature.

[8] Yoshido A., Marec F., Sahara K. (2016). The fate of W chromosomes in hybrids between wild silkmoths, *Samia cynthia* ssp.: No role in sex determination and reproduction. Heredity.

[9] Perrin N. (2009). Sex reversal: A fountain of youth for sex chromosomes?. Evolution.

[10] Schartl M., Schmid M., Nanda I. (2016). Dynamics of vertebrate sex chromosome evolution: From equal size to giants and dwarfs. Chromosoma.

[11] Bachtrog D. (2013). Y-chromosome evolution: Emerging insights into processes of Y-chromosome degeneration. Nat. Rev. Genet..

[12] Skaletsky H., Kuroda-Kawaguchi T., Minx P.J., Cordum H.S., Hillier L., Brown L.G., Repping S., Pyntikova T., Ali J., Bieri T. (2003). The male-specific region of the human Y chromosome is a mosaic of discrete sequence classes. Nature.

[13] Soh Y.Q.S., Alföldi J., Pyntikova T., Brown L.G., Graves T., Minx P.J., Fulton R.S., Kremitzki C., Koutseva N., Mueller J.L. (2014). Sequencing the mouse Y chromosome reveals convergent gene acquisition and amplification on both sex chromosomes. Cell.

[14] Charlesworth B., Crow J.F. (1978). Model for evolution of Y chromosomes and dosage compensation. Proc. Natl. Acad. Sci. USA.

[15] Tomaszkiewicz M., Rangavittal S., Cechova M., Sanchez C., Fescemyer H.W., Harris R., Ye D., Brien C.M.O., Chikhi R., Ryder O.A. (2016). A time- and cost-effective strategy to sequence mammalian Y chromosomes: An application to the *de novo* assembly of gorilla Y. Genome Res..

[16] Hughes J.F., Skaletsky H., Pyntikova T., Graves T.A., Van Daalen S.K.M., Minx P.J., Fulton R.S., McGrath S.D., Locke D.P., Friedman C. (2010). Chimpanzee and human Y chromosomes are remarkably divergent in structure and gene content. Nature.

[17] Kichigin I.G., Giovannotti M., Makunin A.I., Ng B.L., Kabilov M.R., Tupikin A.E., Barucchi V.C., Splendiani A., Ruggeri P., Rens W. (2016). Evolutionary dynamics of *Anolis* sex chromosomes revealed by sequencing of flow sorting-derived microchromosome-specific DNA. Mol. Genet. Genom..

[18] Traut W., Vogel H., Glöckner G., Hartmann E., Heckel D.G. (2013). High-throughput sequencing of a single chromosome: A moth W chromosome. Chromosom. Res..

[19] Wang J., Na J., Yu Q., Gschwend A.R., Han J., Zeng F., Aryal R., VanBuren R., Murray J.E., Zhang W. (2012). Sequencing papaya X and Y^h^ chromosomes reveals molecular basis of incipient sex chromosome evolution. Proc. Natl. Acad. Sci. USA.

[20] Beaudry F.E.G., Barrett S.C.H., Wright S.I. (2017). Genomic loss and silencing on the Y chromosomes of *Rumex*. Genome Biol. Evol..

[21] Crowson D., Barrett S.C.H., Wright S.I. (2018). Purifying and positive selection influence patterns of gene loss and gene expression in the evolution of a plant sex chromosome system. Mol. Biol. Evol..

[22] Pucholt P., Wright A.E., Conze L.L., Mank J.E., Berlin S. (2017). Recent sex chromosome divergence despite ancient dioecy in the willow *Salix viminalis*. Mol. Biol. Evol..

[23] Fontaine A., Filipović I., Fansiri T., Hoffmann A.A., Cheng C., Kirkpatrick M., Rašić G., Lambrechts L. (2017). Extensive genetic differentiation between homomorphic sex chromosomes in the mosquito vector, *Aedes aegypti*. Genome Biol. Evol..

[24] Lambert M.R., Skelly D.K., Ezaz T. (2016). Sex-linked markers in the North American green frog (*Rana clamitans*) developed using DArTseq provide early insight into sex chromosome evolution. BMC Genom..

[25] Conte M.A., Gammerdinger W.J., Bartie K.L., Penman D.J., Kocher T.D. (2017). A high quality assembly of the Nile Tilapia (*Oreochromis niloticus*) genome reveals the structure of two sex determination regions. BMC Genom..

[26] Reichwald K., Petzold A., Koch P., Downie B.R., Hartmann N., Pietsch S., Baumgart M., Chalopin D., Felder M., Bens M. (2015). Insights into sex chromosome evolution and aging from the genome of a short-lived fish. Cell.

[27] Zhang A., Huang R., Chen L., Xiong L., He L., Li Y., Liao L., Zhu Z., Wang Y. (2017). Computational identification of Y-linked markers and genes in the grass carp genome by using a pool-and-sequence method. Sci. Rep..

[28] Liu H., Pang M., Yu X., Zhou Y., Tong J., Fu B. (2018). Sex-specific markers developed by next-generation sequencing confirmed an XX/XY sex determination system in bighead carp (*Hypophthalmichehys nobilis*) and silver carp (*Hypophthalmichthys molitrix*). DNA Res..

[29] Li S., Ajimura M., Chen Z., Liu J., Chen E., Guo H., Tadapatri V., Reddy C.G., Zhang J., Kishino H. (2018). A new approach for comprehensively describing heterogametic sex chromosomes. DNA Res..

[30] Meredith R.W., Pires M.N., Reznick D.N., Springer M.S. (2010). Molecular phylogenetic relationships and the evolution of the placenta in *Poecilia* (*Micropoecilia*) (Poeciliidae: Cyprinodontiformes). Mol. Phylogenet. Evol..

[31] Nanda I., Schories S., Tripathi N., Dreyer C., Haaf T., Schmid M., Schartl M. (2014). Sex chromosome polymorphism in guppies. Chromosoma.

[32] Wright A.E., Darolti I., Bloch N.I., Oostra V., Sandkam B., Buechel S.D., Kolm N., Breden F., Vicoso B., Mank J.E. (2017). Convergent recombination suppression suggests role of sexual selection in guppy sex chromosome formation. Nat. Commun..

[33] Lindholm A., Breden F. (2002). Sex chromosomes and sexual selection in Poeciliid fishes. Am. Nat..

[34] Winge Ö. (1927). The location of eighteen genes in *Lebistes reticulatus*. J. Genet..

[35] Rangavittal S., Harris R.S., Cechova M., Tomaszkiewicz M., Chikhi R., Makova K.D., Medvedev P. (2017). RecoverY: *K*-mer-based read classification for Y-chromosome-specific sequencing and assembly. Bioinformatics.

[36] Carvalho A., Clark A. (2013). Efficient identification of Y chromosome sequences in the human and *Drosophila* genomes. Genome Res..

[37] Akagi T., Henry I.M., Tao R., Comai L. (2014). A Y-chromosome-encoded small RNA acts as a sex determinant in persimmons. Science.

[38] Torres M.F., Mathew L.S., Ahmed I., Al-azwani I.K., Rivera D., Mohamoud Y.A., Clark A.G., Malek J.A. (2018). Genus-wide sequencing supports a two-locus model for sex-determination in *Phoenix*. bioRxiv.

[39] Tomaszkiewicz M., Medvedev P., Makova K.D. (2017). Y and W chromosome assemblies: Approaches and discoveries. Trends Genet..

[40] Tripathi N., Hoffmann M., Willing E.-M., Lanz C., Weigel D., Dreyer C. (2009). Genetic linkage map of the guppy, *Poecilia reticulata*, and quantitative trait loci analysis of male size and colour variation. Proc. Biol. Sci..

[41] Tripathi N., Hoffmann M., Weigel D., Dreyer C. (2009). Linkage analysis reveals the independent origin of poeciliid sex chromosomes and a case of atypical sex inheritance in the guppy (*Poecilia reticulata*). Genetics.

[42] Bolger A.M., Lohse M., Usadel B. (2014). Trimmomatic: A flexible trimmer for Illumina sequence data. Bioinformatics.

[43] Song L., Florea L., Langmead B. (2014). Lighter: Fast and memory-efficient error correction without counting. Genome Biol..

[44] Marçais G., Kingsford C. (2011). A fast, lock-free approach for efficient parallel counting of occurrences of *k*-mers. Bioinformatics.

[45] Simpson J.T., Wong K., Jackman S.D., Schein J.E., Jones S.J.M. (2009). ABySS: A parallel assembler for short read sequence data. Genome Res..

[46] Altschup S.F., Gish W., Miller W., Myers E.W., Lipman D.J. (1990). Basic Local Alignment Search Tool. J. Mol. Biol..

[47] Ku A., Hoffmann M., Fraser B.A., Kottler V.A., Sharma E., Weigel D., Dreyer C. (2016). The genome of the Trinidadian guppy, *Poecilia reticulata*, and variation in the Guanapo population. PLoS ONE.

[48] Carvalho A.B., Vicoso B., Russo C.A.M., Swenor B., Clark A.G. (2015). Birth of a new gene on the Y chromosome of *Drosophila melanogaster*. Proc. Natl. Acad. Sci. USA.

[49] Bull J.J. (1983). Evolution of Sex Determining Mechanisms.

[50] Hall A.B., Qi Y., Timoshevskiy V., Sharakhova M.V., Sharakhov I.V., Tu Z. (2013). Six novel Y chromosome genes in *Anopheles* mosquitoes discovered by independently sequencing males and females. BMC Genom..

[51] Meisel R., Gonzales C.A., Luu H. (2017). The house fly Y chromosome is young and undifferentiated from its ancient X chromosome partner. Genome Res..

[52] VanKuren N.W., Long M. (2018). Gene duplicates resolving sexual conflict rapidly evolved essential gametogenesis functions. Nat. Ecol. Evol..

[53] Nanda I., Kondo M., Hornung U., Asakawa S., Winkler C., Shimizu A., Shan Z., Haaf T., Shimizu N., Shima A. (2002). A duplicated copy of *DMRT1* in the sex-determining region of the Y chromosome of the medaka, *Oryzias latipes*. Proc. Natl. Acad. Sci. USA.

[54] Hattori R.S., Murai Y., Oura M., Masuda S., Majhi S.K., Sakamoto T., Fernandino J.I., Somoza G.M., Yokota M., Strussmann C.A. (2012). A Y-linked anti-Mullerian hormone duplication takes over a critical role in sex determination. Proc. Natl. Acad. Sci. USA.

[55] Gallach M., Betrán E. (2011). Intralocus sexual conflict resolved through gene duplication. Trends Ecol. Evol..

[56] Grossen C., Neuenschwander S., Perrin N. (2012). The evolution of XY recombination: Sexually antagonistic selection versus deleterious mutation load. Evolution.

[57] Parichy D.M., Rawls J.F., Pratt S.J., Whitfield T.T., Johnson S.L. (1999). Zebrafish sparse corresponds to an orthologue of c-kit and is required for the morphogenesis of a subpopulation of melanocytes, but is not essential for hematopoiesis or primordial germ cell development. Development.

[58] Kottler V.A., Fadeev A., Weige D., Dreyer C. (2013). Pigment pattern formation in the guppy, *Poecilia reticulata,* involves the Kita and *Csf1ra* receptor tyrosine kinases. Genetics.

[59] Fisher R.A. (1931). The evolution of dominance. Biol. Rev..

[60] Hiort O. (2013). The differential role of androgens in early human sex development. BMC Med..

[61] Oike A., Kodama M., Yasumasu S., Yamamoto T., Nakamura Y., Ito E., Nakamura M. (2017). Participation of androgen and its receptor in sex determination of an amphibian species. PLoS ONE.

[62] Uno Y., Hosaka S., Yamazaki H. (2014). Identification and analysis of *CYP27A, CYP17A1, CYP20A1, CYP27A1* and *CYP51A1* in *Cynomolgus* Macaques. J. Vet. Med. Sci..

[63] Mast N., Lin J.B., Pikuleva I.A. (2015). Marketed drugs can inhibit cytochrome p450 27A1, a potential new target for breast cancer adjuvant therapy. Mol. Pharmacol..

[64] Kohno S., Bernhard M.C., Katsu Y., Zhu J., Bryan T.A., Doheny B.M., Iguchi T., Guillette L.J. (2015). Estrogen receptor 1 (ESR1; ERα), not ESR2 (ERβ), modulates estrogen-induced sex reversal in the American alligator, a species with temperature-dependent sex determination. Endocrinology.

[65] Maruo K., Suda M., Yokoyama S., Oshima Y., Nakamura M. (2008). Steroidogenic gene expression during sex determination in the frog *Rana rugosa*. Gen. Comp. Endocrinol..

[66] Nakamura M. (2012). Is a sex-determining gene(s) necessary for sex-determination in amphibians? Steroid hormones may be the key factor. Sex. Dev..

[67] Kong X.-F., Li J.-T., Sun X.-W. (2016). Complete mitochondrial genome of the guppy (*Poecilia reticulata)*. Mitochondrial DNA.

