# Supplementary figures and images for: Shared and Species-Specific Patterns of Nascent Y Chromosome Evolution in Two Guppy Species
Source: Genes (Basel). 2018 May 3;9(5):238. doi: 10.3390/genes9050238 (PMC5977178; doi:10.3390/genes9050238)

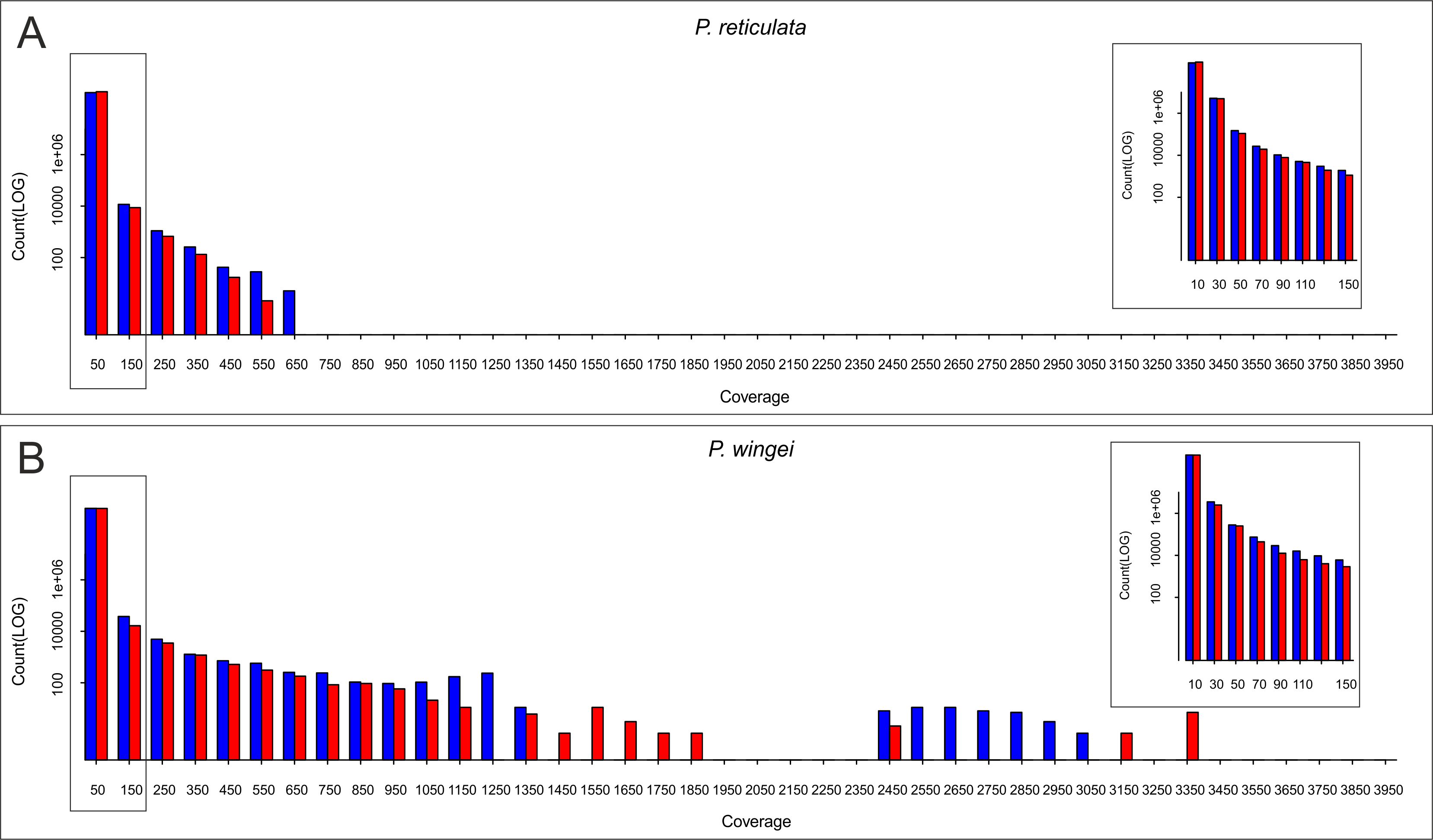

Supplement: Supplementary file 1 [file genes-09-00238-s001.zip › Guppys_KMer_Supplementary/Guppys_KMer_SFig1.tif]
